# High Prevalence of Malnutrition among the Above Thirteen with Primary Pyomyositis in Northern Uganda

**DOI:** 10.9734/BJMMR/2015/14597

**Published:** 2015

**Authors:** David Lagoro Kitara, Paul Okot Bwangamoi, Henry Wabinga, Michael Odida

**Affiliations:** 1 Department of Surgery, Faculty of Medicine, Gulu University, Gulu, Uganda.; 2 Department of Pharmacy, Faculty of Medicine, Gulu University, Gulu, Uganda.; 3 Department of Pathology, College of Health Sciences, Makerere University, Uganda.

**Keywords:** High prevalence, malnutrition, primary pyomyositis, Uganda

## Abstract

**Aim:**

To determine the prevalence of malnutrition and its association with primary pyomyositis among patients and controls who were age and sex matched.

**Study Design and Setting:**

A case-control study was conducted at Gulu Regional, Lacor, Kalongo, Kitgum and St. Joseph’s Hospitals in Northern Uganda.

**Study Duration:**

Study was conducted from September 2011 to November 2013.

**Methods:**

Primary pyomyositis patients were consecutively recruited to these Hospitals and were age and sex-matched with controls selected during the same period. History, physical examinations, Body Mass Index (BMI), blood samples for haematology, biochemistry, clinical chemistry and muscle biopsy for histology were obtained. Those that did not meet the inclusion criteria were excluded. The study was approved by the Ethics and Review Committee of Gulu University Medical School.

**Results:**

During the study period, 63 patients and 63 controls were recruited; 29 females and 34 males. Among primary pyomyositis patients, 59 (93.7%) had malnutrition while there were 2 in the control group, giving a prevalence of 3.2%.The matched analysis produced an aOR of 449.875 with a 95% CI (79.382, 2549.540; p<0.001) for malnutrition. Among the cases, 16 (25.4%) fulfilled the Clinical Case Definition (CCD) for AIDS, compared to 2 (3.2%) among the controls. The adjusted Odds ratio for the difference in fulfilling the CCD for AIDS between cases and controls was statistically significant aOR of 10.383 with a 95% CI (2.275, 47.397; p<0.001).

**Conclusion:**

Primary pyomyositis is a common health problem in Northern Uganda. It is evident that malnutrition is the most common risk factor in Primary pyomyositis especially among the above thirteen year olds in Northern Uganda.

## 1. INTRODUCTION

Pyomyositis is a suppurative inflammation of large truncal and limb muscles and in most cases it is caused by *Staphylococcus aureus* [1]. It is an infection which is increasingly common in non-tropical regions but has been highly prevalent in tropical regions [2]. Although many cases of pyomyositis has been reported in patients with immunosuppression including HIV/AIDS since 1987 [3], few studies have been conducted to confirm the role of malnutrition as a risk factor for primary pyomyositis especially in Northern Uganda [4]. The risk factors for the development of pyomyositis has however been described to include: immunodeficiency, trauma to muscle, injection drug use, and concurrent infection [1, 3, 5, 6, 7, 8]. Immunodeficiency especially resulting from HIV/AIDS has been implicated in the development of pyomyositis in both temperate and tropical climates [1, 9]. Studies have shown that the other common forms of immunodeficiency associated with pyomyositis include Diabetes mellitus, malignancy, renal insufficiency, organ transplantation and administration of immunosuppressive agents [1, 9, 10]. These immunosuppressive conditions have been found to be particularly important risk factors [1, 2, 7, 11, 12, 13, 14] in the pathogenesis of pyomyositis and were recognized in 1948 by Burkitt [15, 16].

Pyomyositis has been found mainly in the sexually active age groups and a significant proportion of the patients had been exposed to the risk factors for HIV infection [17]. Most patients who present with pyomyositis are HIV positive and are mostly suffering from AIDS-related complexes [15, 17, 18, 19].

Other hypotheses have connected demographic factors associated with pyomyositis to such tropically predisposed circumstances such as septicaemia [20, 21], protozoan infections [22, 23], viral muscular infections [24] and disordered immunity [15, 25].

Reports from Northern Uganda indicate that pyomyositis is a very common disease and it is a major cause of severe disability and morbidity that contributes significantly to prolonged hospital stay [4]. Knowledge of the risk factors would help medical personnel to design strategies for the prevention and management of this condition.

In this study, we therefore conducted a case-control comparison of the prevalence and malnutrition as a risk factor (using clinical features, BMI and serum albumin levels) between primary pyomyositis patients and controls to establish the relationship between primary pyomyositis and malnutrition in Northern Uganda.

## 2. PATIENTS AND METHODS

### 2.1 Study Design

A case-control study was conducted between September 2011 and November 2013.

### 2.2 Study Sites

This study was conducted at Gulu Regional, St. Mary’s Hospital Lacor, Kitgum St. Joseph’s, Kitgum Government and Kalongo Hospitals in Northern Uganda. This region is just recovering from over 20 years of civil war in which nearly 90% of the population of about two million were incarcerated into the infamous internally displaced peoples camps (IDPs). During the 10–12 years in the camps, they were fed on food provided by the United Nations World Food program (UNWFP). Reports from the UNWFP indicated that the organization was providing IDPs with food which was just 60% of the required calories per day. This was distributed on a bi-weekly basis. The areas around the camps were so insecure that IDPs could not farm in their own gardens to obtain additional food to supplement those provided by UNWFP. It was reported that the prevalence of malnutrition rose in Northern Uganda during the periods in the camps [4].

Primary pyomyositis was therefore defined as suppurative inflammation of one or more skeletal muscles as primary sites shown by tender swelling, presence of pus and confirmed by histology of muscle biopsy.

The World Health Organization defines malnutrition as the cellular imbalance between supply of nutrients and energy and the body's demand for them to ensure growth, maintenance, and specific functions [26]. In the context of this study, malnutrition was defined as low BMI (<18.5), observed clinical features of malnutrition and low serum albumin of less than 38 g/L.

All patients who satisfied the case definition and controls were enrolled consecutively and evaluated according to the World Health organization (WHO) Clinical Case Definition (CCD) for AIDS [27].

### 2.3 Inclusion Criteria for Cases

Primary pyomyositis patients who were 13 years and above, had provided informed consent/Assent and with histological confirmation of muscle biopsy.

### 2.4 Exclusion Criteria for the Cases

Pyomyositis with suppurative infection in the neighbouring structures.

### 2.5 Inclusion Criteria for Controls

For each patient, an age and sex matched control was selected from an otherwise healthy individual with minor trauma admitted to the surgery unit of these hospitals within 24 hours of injury in the same month of the study period. The age-matched controls were ±2 years and from the same sub county and residents of the hospital catchment areas.

### 2.6 Exclusion Criteria for the Controls

Lack of informed consent and reporting to the health facilities more than 24 hours after injury.

### 2.7 Data Collection

Data on patients and controls were collected using a pretested questionnaire that was designed for the recruitment and follow-up of the study participants. The questionnaire collected the socio-demographic characteristics of participants and risk factors of primary pyomyositis. The information obtained was kept under lock and key in the faculty of Medicine of Gulu University and only accessed by the Principal Investigator of this study.

### 2.8 Clinical Assessment

Each case and control underwent a clinical evaluation to assess the features of malnutrition right from head to toe; the hair distribution and its texture, the body stature, skin textures and thickness, the mouth, nails and the mucous membrane for pallor.

### 2.9 Anthropometric Measurements

For each case and control, weight was measured using a standardized weighing scale and measured to (0.1Kg). Height was measured when the participant was standing in an erect position, bare footed on a stadiometer with a movable head piece. The head piece was leveled with skull vault & height was recorded to the nearest 0.5 cm. The BMI was then calculated using the formula BMI=weight (Kg)/Height^2^ (Meter^2^). Each person’s BMI was then graded according to the WHO (2007) classification to categorize it into: BMI <18.5 as Under Weight; BMI 18.5–24.5 as Healthy weight range; BMI 25–30 as Overweight (grade 1 obesity); BMI >30–40 as Obese (grade 2 obesity); BMI >40 as Very obese (morbid or grade 3 obesity) [28].

### 2.10 Laboratory Procedures

Blood samples were obtained from the cubital fossae of each case and control using aseptic technique and stored in 2 separate bottles (EDTA and plain). Those in the plain sterile bottles were centrifuged at 1500 revolutions per minute to obtain serum for biochemical test and transferred into cryo-vials and stored at −20°C before the biochemical analysis for serum albumin level were conducted. A value less than 38 g/L was considered low serum albumin and therefore compared with the BMI and clinical features to confirm the presence of malnutrition. The same serum was controlled in another ISO accredited laboratory using the same method of serum analysis for albumin. Haematological analysis was conducted on the blood samples collected in the EDTA container to obtain the haemoglobin concentration and those found less than 10 g/dl were considered low haemoglobin and therefore anaemic. Muscle biopsy was taken from each case for histological analysis and results used for confirmation of a diagnosis as primary or secondary pyomyositis.

### 2.11 Ethical Consideration

The study was approved by the Research and Ethics Committee of Gulu University Medical School and Uganda National Council of Science and Technology (UNCS&T) number HS 922 and the research was conducted in accordance with the principles of good clinical practice and standards. All parents/guardians of cases and controls for those below the consenting age gave a written informed consent. Confidentiality of information obtained was maintained throughout the study and follow-up of the cases.

### 2.12 Data Analysis

The statistical software package, SPSS version 15.0 (Chicago, IL, USA) was used for the univariate analysis of socio-demographic characteristics and other variables. Bivariate analysis was used to test the associations between the outcome and independent variables. Odds Ratios (OR) with a 95% Confidence Interval (CI) was calculated to determine the risk factors between cases and controls. Fisher’s exact t–test was used where cell numbers were less than five. A multivariable regression analysis was used to determine the risk factors for primary pyomyositis. A p-value of less than 0.05 was used as the cut off for the level of statistical significance.

## 3. RESULTS

Table 1 shows that there is no association between socio-demographic characteristics and the occurrence of primary pyomyositis.

Table 2 shows that there are some clinical features which are statistically and significantly associated with the occurrence of primary pyomyositis

Table 3 shows that features of immunosuppression especially AIDS, low haemoglobin concentration, high creatinine level, low serum albumin level, low CD_4_/CD_8_ counts and the presence of Hookworms in stool were key risk factors for primary pyomyositis in patients in Northern Uganda

**Fig. 1:**
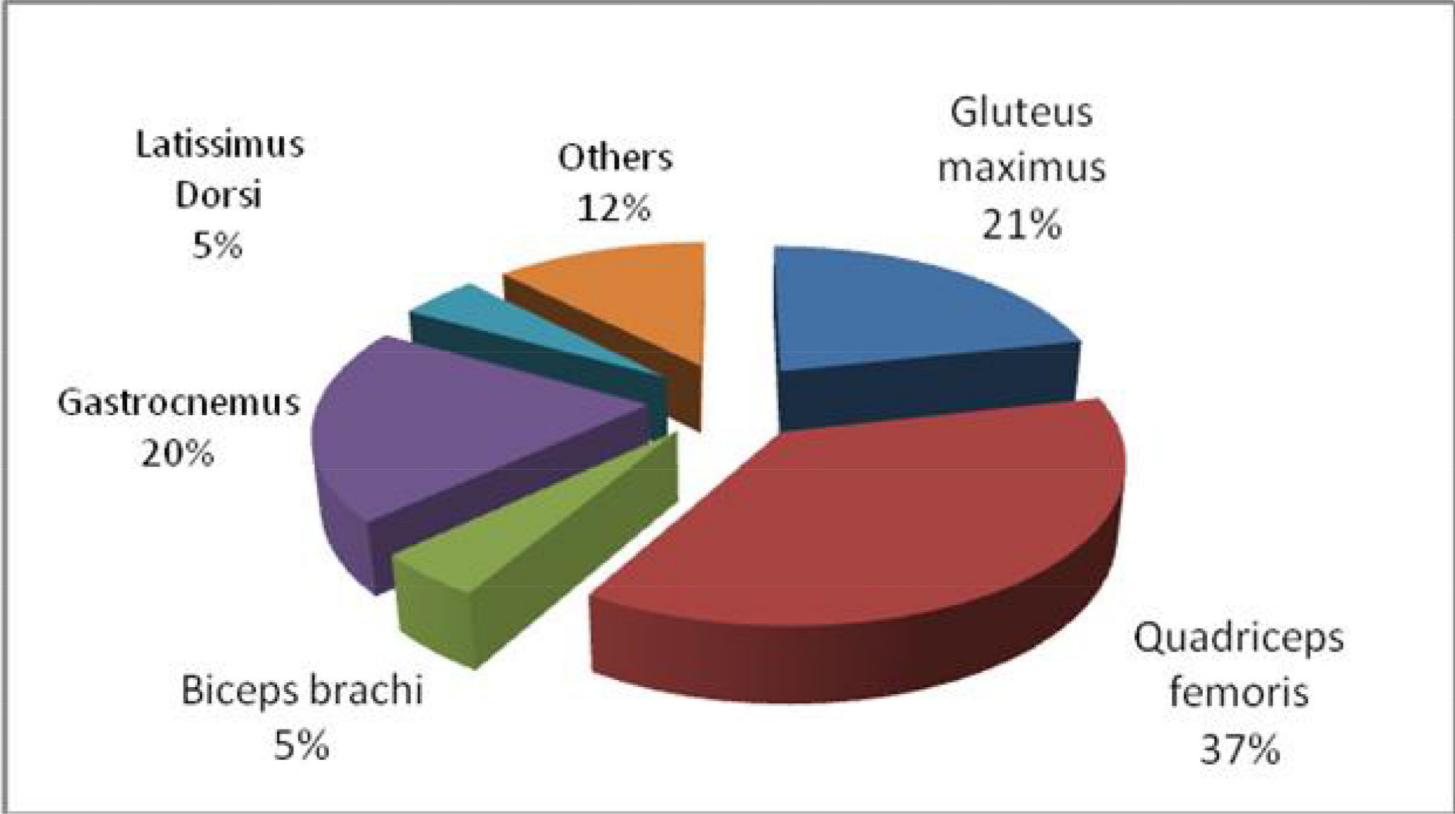
Shows the muscles involved in primary pyomyositis

Fig. 1 shows that the most commonly affected muscles were quadriceps femoris, gluteus maximus, gastrocnemus and biceps brachi in descending order respectively.

During the study period, 63 primary pyomyositis patients were admitted to Hospitals in Northern Uganda. The mean age of the patients was 22 years (SD±10.667). The patients were divided in the different age groups as follows: 29 in the second decade, 18 in the third decade, 9 in the fourth decade, 5 in the fifth and above decades of life. The female to male ratio was 1:1.2 (29 female and 34 males) (Table 1). Out of these 63 patients, 5 (7.9%) had multiple muscular lesions: Two lesions were observed in three patients while three in two patients. Fifty nine patients (93.7%) had malnutrition; while the controls had two with a prevalence of (3.6%); one in the second decade and another in the third decade of life.

### 3.1 The Muscles Involved

This study found that the most commonly affected muscles with primary pyomyositis were Quadriceps femoris 24 (34.8%), Gluteus maximus 14(20.3%), Gastrocnemus 13(18.84%); *Latissimus dorsi* 3 (4.35%); Biceps brachi 3 (4.35%) and others respectively (Fig. 1).

### 3.2 Histology Findings

The muscles involved were necrotic with some parts containing muscle tissues that had undergone fibrous degeneration. There was mass infiltration of the tissue with cells of immune response, plenty of pus cells interspersed with giant cells. The blood vessels were collapsed and filled with thrombus and some had undergone fibrous degeneration.

### 3.3 The Clinical Features

Most primary pyomyositis patients had clinical features of malnutrition characterized by low body weight, slim stature, poor hair distribution with silky hair, poor skin colour, texture and thin skin fold. The nails where brittle and some with a spoonlike appearance; the skin were scaly with evident bone prominences. The mucous membranes were pale in most patients.

The matched analysis for malnutrition produced an adjusted Odds ratio of 449.875 with a 95% CI of (79.382, 2549.540). All patients with multiple muscular lesions had features of malnutrition and were all HIV negative. The differences in the prevalence of malnutrition between cases and controls was statistically significant (p<0.001) (Table 3). Cases and controls were evaluated on a scale for WHO Clinical Case Definition (CCD) for AIDS. There were 16 (25.4%) primary pyomyositis patients that met these criteria: Of these 8 (72.2%) were HIV positive and 8 (15.4%) were HIV negative compared with 2 (3.2%) in the control group who were all HIV negative. The difference in fulfilling the WHO (CCD) for AIDS between study participants produced a statistically significant result (p<0.001) with an adjusted Odds ratio of 10.383 with a 95% CI of (2.275, 47.397) (Table 3).

All primary pyomyositis patients underwent Incision, Drainage & Debridement (I,D&D); a procedure that was able to evacuate pus and necrotic muscle tissue. They were treated with antibiotics on the basis of the antibiotic susceptibility results. The commonest organisms cultured were: *Staphylococcus aureus* (95%); *Escherichia coli* (2.5%) and *streptococcus pyogenes* (2.5%). ZN staining of pus sample was also conducted and there was no Acid Alcohol Fast Bacilli (AAFBs) observed. *Staphylococcus aureus* was susceptible to Tetracycline, Ciprofloxacin, Erythromycin, Methicillin and Gentamycin. It was however resistant to co-trimoxazole. All patients discharged in an improved condition after a median duration of Hospital stay of 10.95 days (SD±3.761).

## 4. DISCUSSION

Pyomyositis is a common clinical entity in Northern Uganda and it affects large numbers of people. It is locally known as “Two rec” which is translated as, “the disease which affects muscle and the muscle colour is whitish like the flesh of fish”. The socio-demographic characteristics of primary pyomyositis patients were comparable to most studies conducted in the tropical region [4] (Table 1). Large proximal lower limb and truncal muscles were the most commonly affected and the most commonly isolated organism was *Staphylococcus aureus* [4] (Fig. 1).

Pyomyositis has been considered primarily a disease of the tropics that occurs mainly in young and relatively healthy persons [29, 30]. Several unproven hypotheses have been proposed to explain this disorder including malnutrition, HIV/AIDS, protozoa infection, viral muscle infection, trauma and disordered immunity [2, 3, 29, 31, 32, 33, 34]. It has been previously noted that muscles were normally remarkably resistant to suppurative infections and muscle trauma has been reported to be necessary before an experimentally induced bacteriaemia could cause pyomyositis in animals [35]. In humans, muscle abscesses were rarely a complication of severe staphylococcal sepsis [36]. This finding was perhaps an indication that trauma alone was not sufficient to cause pyomyositis.

In this study, information derived from these primary pyomyositis patients adds more information to previous studies from tropical regions which revealed that pyomyositis patients were generally young adults with few reported or confirmed cases of Diabetes mellitus or bleeding disorders but that the majority of the patients were generally malnourished. Even those patients that had other risk factors, the commonest denominator to this disease in Northern Uganda was malnutrition which was shown by low serum albumin level (<38 g/L), low BMI (<18.5) and with overt clinical features of malnutrition (Tables 2 and 3).

HIV/AIDS infection has been reported as a common finding in patients with primary pyomyositis and it is a risk factor which makes muscles of these patients susceptible to bacterial infection [1, 11]. For HIV positive primary pyomyositis patients, 8/11 (72.7%) had malnutrition; a factor which was perhaps important in the epidemiology of this disease. Not all HIV positive patients have an increased risk of developing pyomyositis but rather those with low CD_4_ counts of less than 250 cells per ml that developed pyomyositis. For HIV negative patients, some had malnutrition clinically to a level that 8/52 (15.4%) met the WHO criteria for Clinical Case Definition (CCD) of AIDS; an indication that malnourished primary pyomyositis patients in this region were so grossly affected to such a great level that they could be described as AIDS patients; a presentation that was once described as the “slim” disease in Uganda in the early 1980s (Table 3).

Although primary pyomyositis patients in this study had other underlying conditions that may have predisposed them to the development of the disease example trauma, HIV/AIDS, bleeding disorders, chronic alcoholic intake, Diabetes mellitus, and low haemoglobin concentration (Table 2), the result presented has demonstrated that primary pyomyositis is statistically and significantly associated more with malnutrition. The increased incidence of primary pyomyositis in persons with malnutrition appears to occur perhaps as a result of an increased rate of asymptomatic *Staphylococcus aureus* infection [37] or dysfunctional/inadequate numbers of cells for immune defense thus allowing higher rates of staphylococcus infection and bacteriaemia [38]. It is also important to note here that whereas, Tuberculosis is a very common communicable disease in tropical regions especially with the advent of widespread HIV/AIDS, none of these patients were observed with tuberculous pyomyositis.

The study participants in this study were persons thirteen years and above. This age limited a number of those below thirteen years from being participants and therefore, this exclusion of some of the likely study participants may be a limiting factor for this particular study however it may also provide an opportunity for future studies in which persons below 13 years could be examined separately to observe whether there are any differences in the findings from what we have observed in this study.

## 5. CONCLUSION

Primary pyomyositis is a common health problem in Northern Uganda. It is evident that malnutrition is the most common risk factor in Primary pyomyositis especially among young people above thirteen years in Northern Uganda.

## Figures and Tables

**Table 1:** Socio-demographic characteristics and its association with malnutrition

Ages (yrs)	Freq	%	p-value	(aOR)	95%CI
10–19	29	46.03			
20–29	18	28.57			
30–39	11	17.46	0.243	3.214	0.414, 24.96
40–49	3	4.76			
50–59	1	1.59			
60+	1	1.59			
**Sex**					
Male	34	53.97	0.383	2.71	0.266, 27.577
Female	29	46.03			
**Tribes**					
Acholi	58	92.06			
Lango	4	6.35	0.544	0.931	0.868, 0.999
Madi	1	1.59			
**Marital status**					
Married	28	44.44			
Single	33	53.38	0.383	2.71	0.266, 27.577
Co-Habiting	1	1.59			
Separated	1	1.59			
**Religion**					
Protestant	11	17.46			
Catholic	45	71.43	0.87	1.214	0.118, 12.577
Born Again	7	11.11			
**Duration hospital of (days) stay**					
1–7 days	11	17.46	0.23	3.808	0.374, 38.777
8–14 days	45	71.43			
15–21 days	7	11.11			
**Occupation**					
Pupil	20	31.75			
Student	12	19.05			
Business	4	6.35	0.317	3.103	0.305, 31.580
Teacher	1	1.59			
Housewife	2	3.17			
Peasant farmer	24	38.1			
**Highest level of education attained**					
Nil	2	3.17			
Primary	41	65.08	0.465	2.105	0.275, 16.104
Secondary	19	30.16			
Diploma	1	1.59			

**Table 2:** Other different presentations of primary pyomyositis

Clinical characteristics	p-value	(aOR)	95% CI	Fisher’s test
History of trauma to the muscle	0.035	7.429	0.898, 61.447	0.094
Features of immunosuppression/AIDS	0.228	1.093	1.002, 1.193	0.299
Previous pyomyositis	0.793	1.069	1.001, 1.141	1.000
Bleeding disorder	0.708	1.07	1.001, 1.144	1.000
**Laboratory findings**				
HIV negative	0.681	1.633	0.154, 17.353	0.546
Low haemoglobin concentration	0.056	1.133	1.002, 1.281	0.118
Abnormal Creatinine	0.714	1.458	0.192, 11.078	
Low CD_4_/CD_8_ counts	<0.001	6.5	1.817, 23.258	
Positive stool exam	0.292	1.087	1.002, 1.180	

**Table 3:** Shows the case-control results between pyomyositis patients and their controls

Variables	Cases (n=63)	Controls (n=63)	p-value	Adj(OR)	95% CI
Ages (<30 yrs)	47	51	0.391	0.691	0.296, 1612
Sex(female)	29	29	1.000	1.000	0.496, 2.015
Features of immunosuppression/AIDS	16	2	<0.001	10.383	2.275, 47.397
Low haemoglobin	29	4	<0.001	12.	4.075, 38.843
High Creatinine level	37	28	0.001	8.429	4.205, 16.890
Low serum albumin level	59	2	<0.001	449.875	79.382, 2549.540
HIV negative	52	53	0.811	0.892	0.349, 2.279
HIV positive	11	10	0.811	1.121	0.439, 2.864
Low CD_4_/CD_8_ counts	8	0	0.001	4.333	1.606, 11.691
Hookworms in stool	7	0	0.002	1.087	1.002, 1.180

Serum creatinine (Normal = <0.7 mmol/L); HIV=Human immunodeficiency virus; Hb=Haemoglobin concentration (normal> 10 g/dl); CD=Cluster of Differentiation (Low CD_4_ Count = <250 cells/ml); Low serum albumin (<38 g/L)
